# Assessment of the information quality of chatbot technologies on orthodontic miniscrews

**DOI:** 10.1590/2177-6709.30.5.e2524255.oar

**Published:** 2025-12-01

**Authors:** Başak Baş YAMAÇ, Rojda AKÇAR, İpek Eryılmaz ŞARKAN, Can ARSLAN

**Affiliations:** 1 Istanbul Medipol University, Faculty of Dentistry, Department of Orthodontics (Istanbul, Turkey).; 2 Yeditepe University, Faculty of Dentistry, Department of Orthodontics (Istanbul, Turkey).

**Keywords:** Artificial Iintelligence, Large language models, TAD, ChatGPT, Inteligência artificial, Modelos de Linguagem de Grande Escala, DAT, ChatGPT

## Abstract

**Introduction:**

In this era of artificial intelligence, the increasing competition has significantly enhanced the power of AI chatbots, which have been integrated into various fields, including orthodontics. They can be a good option for enlightening topics of curiosity before or during treatment, as they are easily accessible by patients. However, their reliability remains a concern. Miniscrews, or Temporary Anchorage Devices (TADs), frequently used during orthodontic treatments, are among the subjects of interest to patients.

**Objective:**

This study aimed to comparatively evaluate the responses provided by four Large Language Models (LLMs), namely ChatGPT-3.5, ChatGPT-4 (OpenAI), Google Bard (Google LLC), and Bing Chat (Microsoft Corp), to questions asked by patients in the field of orthodontic miniscrews.

**Material and Methods:**

The most frequently asked questions by patients about miniscrews used in orthodontics were searched on Google. The first 50 pages were reviewed, and 30 questions were selected and asked to the LLM. The responses from the LLMs were evaluated using a five point modified scale and modified DISCERN (mDISCERN) by three orthodontic residents.

**Results:**

It was observed that the highest score for Likert belonged to GPT-4 (3.84), while the lowest was for Bing Chat (3.37). Statistically significant differences were found between the median score values given to the questions by all three researchers, depending on the LLMs used (p = 0.016; p<0.001 and 0.017, respectively). Significant differences were found between the scores given by Investigator 1 and Investigator 3 to ChatGPT-4 and Google Bard and the scores given to Bing Chat. Additionally, Investigator 2 showed significant differences in the scores given to Bing Chat compared to ChatGPT-3.5 (p=0.018). Among the evaluated chatbots, ChatGPT-4 achieved the highest mDISCERN score (23.43 ± 2.89), followed by Google Bard (23.47 ± 3.2), ChatGPT-3.5 (22.62 ± 2.97), and Bing Chat (21.63 ± 2.43).

**Conclusions:**

In this study, it was found that LLMs can generally inform patients about miniscrews used in orthodontics and have promising potential. However, it is necessary that the information provided by these programs should always be supported by the information given by professionals.

## RESUMO

**Introdução:**

Nesta era da inteligência artificial, a crescente concorrência aumentou significativamente o poder dos chatbots de IA, os quais foram integrados em várias áreas, incluindo a Ortodontia. Eles podem ser uma boa opção para esclarecer temas de curiosidade antes ou durante o tratamento, pois são facilmente acessíveis pelos pacientes. No entanto, sua confiabilidade continua sendo uma preocupação. Os mini-implantes, ou dispositivos de ancoragem temporária (DATs), frequentemente usados durante os tratamentos ortodônticos, estão entre os assuntos de interesse dos pacientes.

**Objetivo:**

Este estudo teve como objetivo avaliar comparativamente as respostas fornecidas por quatro Modelos de Linguagem de Grande Escala (LLMs), ChatGPT-3.5, ChatGPT-4 (OpenAI), Google Bard (Google LLC) e Bing Chat (Microsoft Corp), a perguntas feitas por pacientes sobre o tema mini-implantes ortodônticos.

**Material e Métodos:**

As perguntas mais frequentes dos pacientes sobre mini-implantes utilizados em Ortodontia foram pesquisadas no Google. As primeiras 50 páginas foram analisadas e 30 perguntas foram selecionadas e enviadas aos LLMs. Três residentes em Ortodontia avaliaram as respostas dos LLMs utilizando uma escala modificada de cinco pontos e o DISCERN modificado (mDISCERN).

**Resultados:**

Observou-se que o escore mais alto para Likert pertencia ao GPT-4 (3,84), enquanto o mais baixo foi para o Bing Chat (3,37). Foram encontradas diferenças estatisticamente significativas entre os valores médios dos escores atribuídos às perguntas pelos três pesquisadores, dependendo dos LLMs utilizados (p = 0,016; p<0,001 e 0,017, respectivamente). Foram encontradas diferenças significativas entre os escores atribuídos pelo Investigador 1 e pelo Investigador 3 ao ChatGPT-4 e ao Google Bard, e os escores atribuídos ao Bing Chat. Além disso, o Investigador 2 apresentou diferenças significativas nos escores atribuídos ao Bing Chat, em comparação com o ChatGPT-3.5 (p=0,018). Entre os chatbots avaliados, o ChatGPT-4 obteve o escore mDISCERN mais alto (23,43 ± 2,89), seguido pelo Google Bard (23,47 ± 3,2), ChatGPT-3.5 (22,62 ± 2,97) e Bing Chat (21,63 ± 2,43).

**Conclusões:**

Neste estudo, verificou-se que os LLMs podem, em geral, informar os pacientes sobre os mini-implantes utilizados em Ortodontia e têm um potencial promissor. No entanto, é necessário que as informações fornecidas por esses programas sejam sempre complementadas pelas informações fornecidas por profissionais.

## INTRODUCTION

John McCarthy used the term artificial intelligence (AI) in 1956 at a conference, to refer to machines that might imitate human knowledge and behaviors. However, the possibility of machines mimicking human behavior and genuinely thinking had been previously mentioned by Alan Turing.^1^ With advancements in computer hardware, AI has gained increased capacity to process large datasets, enabling the simulation of human behavior and the ability to interact with humans.^2^ At present, artificial intelligence (AI) is used in sectors such as production, transportation, energy, financial services, advertising, management, and healthcare.^3^ A significant part of AI technology that has developed notably in recent years is chatbots.^4^ OpenAI (OpenAI, San Francisco, CA, USA) developed ChatGPT (Chat Generative Pre-trained Transformer), which is one of the most advanced chatbots (>175 billion parameters) and was preliminarily released to the market in November 2022. The literature has been trained on a wide range of internet texts (approximately 570 GB), including literature, websites, news, and scientific books.^4^ ^,^ ^5^

In February 2023, Microsoft launched the Bing Chat chatbot using the GPT-4 language model, while in March 2023, Google released the Bard chatbot, initially supported by its own custom LLM family, the Language Model for Dialogue Applications (LaMDA), and later by the Pathways Language Model (PaLM) 2 LLMs. Google Bard offers real-time internet access, in contrast to ChatGPT. OpenAI introduced GPT-4, the latest version of its Large Language Models (LLMs), on March 14, 2023, which is a paid service. ^6^ In the literature, there are studies indicating that GPT-4 provides more creative responses and performs better in carrying out detailed commands compared to GPT-3.5.^7^ ^,^ ^8^

Another chatbot, Bing Chat, is integrated with Microsoft Bing, a search engine. Bing Chat is supported by ChatGPT technology and offers free access to Bing Chat using GPT-4. Although ChatGPT is considered a valuable source of information nowadays, some researchers have expressed concerns about its accuracy, precision, legal implications, and references. ^5^ AI hallucination refers to a situation where AI produces a convincing yet entirely fabricated response. OpenAI’s notes acknowledge that answers generated by ChatGPT may sound reasonable but could be nonsensical or incorrect.^9^

While chatbots are useful in many ways, their applicability in the health sector is an intriguing research topic. Understanding the performance of these different chatbots in the health field can provide insights into which roles each is more suitable for and their strengths and weaknesses.^4^ According to research, ChatGPT has been found to provide highly accurate answers to medical questions.^10^ ^,^ ^11^ Various studies have evaluated the information produced by ChatGPT in fields such as Dental Medicine^12^, Oral and Maxillofacial Surgery^13^, Periodontology^14^, and Orthodontics.^15^

In Orthodontics, anchorage control plays a critical role in effectively managing cases for both structural and facial aesthetics.^16^ ^-^ ^18^ Temporary Anchorage Devices (TADs) offer a valuable alternative method, providing absolute skeletal anchorage and improving orthodontic mechanics.^16^ ^,^ ^19^ ^-^ ^22^ Even if TADs do not pose a financial problem for the patient, the possibility of extra discomfort along with fear can affect the patient’s choice between TADs and other intra/extra-oral orthodontic anchorage appliances. The potential pain and discomfort from additional surgical procedures could be an issue, as it may be the cause of the patient avoiding orthodontic treatment.^23^

Many studies have reported that patients often first prefer the internet for medical information they are curious about.^24^ ^,^ ^25^ The dialogue-based, human-like interaction of ChatGPT and similar LLMs, and their ability to provide meaningful responses to simple requests and questions, make them attractive to users. However, compared to web-based search engines, a major disadvantage of ChatGPT is the impossibility of assessing the reliability of the sources of its responses, and it should not be forgotten that the answers may contain errors.^12^ ^,^ ^26^ With the increasing desire of people to obtain health-related information from LLMs, it is extremely important to provide protection against incorrect answers and misdirections.^12^ Although ChatGPT can be a valuable resource for professionals and patients, its limitations must be recognized.^27^

Thus, the purpose of this study was to evaluate the accuracy of the responses provided by the ChatGPT-3.5, ChatGPT-4, Google Bard, and Bing Chat chatbots when asked questions about miniscrews used in orthodontics.

## MATERIAL AND METHODS

A power analysis conducted using the G*Power 3.1.9.7 software protocol (G*Power, Düsseldorf, Germany)^28^ determined that the total number of questions to be asked to chatbots was 28, with 95% confidence (1-α), 95% test power (1-β), and an effect size of f = 0.884, based on the study by Perez-Pino et al.^15^ Google (California, USA), one of the most commonly used English search engines for simulating real user experience, was used as the primary source for the most frequently asked questions about TADs used in orthodontic treatment. After clearing all previous searches, the phrase *‘Most frequent questions about orthodontic miniscrews?’* was searched on Google, and from the first 50 pages, 30 questions most commonly asked by patients were selected (Table 1). Sponsored links, advertisements, news, and social media websites, non-English websites were excluded.

**Table 1:** Descriptive statistics of the questions asked for the study.

		Likert			mDISCERN		
	Questions	Investigator I (Mean ± SD)	Investigator II (Mean ± SD)	Investigator III (Mean ± SD)	Investigator I (Mean ± SD)	Investigator II (Mean ± SD)	Investigator III (Mean ± SD)
1	What are orthodontic miniscrews?	4.25 ± 0.5	4 ± 0.82	3.5 ± 0.58	23,5 ± 1,29	22 ± 2	22,25 ± 2,5
2	What are the benefits of orthodontic miniscrews?	3.75 ± 0.5	3.25 ± 0.96	3.75 ± 0.5	23,75 ± 5,06	22,75 ± 4,27	24 ± 4,24
3	How are orthodontic miniscrews placed?	3.25 ± 0.5	3.5 ± 1	3.5 ± 0.58	23,75 ± 2,87	24 ± 2,45	24 ± 2,45
4	Do orthodontic miniscrews hurt?	4.25 ± 0.5	4 ± 0	4.5 ± 0.58	25,75 ± 2,06	25,5 ± 1,29	25 ± 1,41
5	How long do orthodontic miniscrews stay in place?	3.5 ± 0.58	3.75 ± 0.5	3.75 ± 0.5	23 ± 0,82	24,75 ± 1,26	25,5 ± 1,73
6	Can orthodontic miniscrews be used with Invisalign?	3.5 ± 1	3.5 ± 1	3.75 ± 0.96	25 ± 1,63	25,25 ± 1,26	26,5 ± 1
7	What are the risks associated with orthodontic miniscrews?	2.75 ± 0.96	3.25 ± 0.5	3.25 ± 0.5	25,75 ± 1,5	25,75 ± 2,87	25,75 ± 2,99
8	How much do orthodontic miniscrews cost?	3 ± 0.82	2.75 ± 0.5	3 ± 0.82	23,5 ± 3,7	23 ± 2,94	23,5 ± 3,42
9	How do I care for my orthodontic miniscrews?	3.5 ± 0.58	3.5 ± 0.58	3.5 ± 0.58	23,25 ± 1,5	23 ± 2,16	22,75 ± 1,71
10	What happens if an orthodontic miniscrew comes loose?	4 ± 0	4 ± 0	4 ± 0	21,75 ± 1,26	21 ± 2	22 ± 2,71
11	Can I eat normally with orthodontic miniscrews?	3.75 ± 0.5	4 ± 0	4 ± 0	23 ± 0,82	23 ± 1,41	23,25 ± 0,5
12	Can I play sports with orthodontic miniscrews?	3.5 ± 0.58	3.5 ± 0.58	3 ± 0.82	24,25 ± 2,22	25,5 ± 3,7	25,75 ± 3,2
13	How do I clean my orthodontic miniscrews?	3.75 ± 0.5	3.75 ± 0.5	3.75 ± 0.5	22,75 ± 0,96	24 ± 0,82	24 ± 2,16
14	What happens after orthodontic miniscrews are removed?	4 ± 0	4 ± 0	4 ± 0	23,25 ± 1,71	23,75 ± 0,96	22,75 ± 1,26
15	Can orthodontic miniscrews be removed easily once the orthodontic treatment is complete?	3.75 ± 0.5	3.5 ± 0.58	3.5 ± 0.58	26,25 ± 3,4	26,25 ± 2,87	25,5 ± 3,32
16	Are orthodontic miniscrews suitable for everyone?	3.75 ± 0.5	3.75 ± 0.5	3.5 ± 0.58	21,75 ± 1,26	22,5 ± 2,38	22 ± 2,71
17	Do orthodontic miniscrews weaken the jawbone?	3.75 ± 0.5	3.5 ± 0.58	3.75 ± 0.5	21,5 ± 1,29	22,75 ± 1,71	23,75 ± 0,96
18	Can orthodontic miniscrews be used to close extraction gaps?	4 ± 0	3.75 ± 0.5	4.25 ± 0.5	24,25 ± 0,5	25,25 ± 0,5	24,75 ± 0,5
19	Are orthodontic miniscrews covered by insurance?	3.75 ± 0.5	3 ± 0	3.25 ± 0.5	23,5 ± 1,29	23,75 ± 0,96	23,5 ± 1,29
20	What are the alternatives to orthodontic miniscrews?	3.5 ± 0.58	3.75 ± 0.5	3.75 ± 0.5	23 ± 2,71	22,5 ± 1,73	23,5 ± 1
21	Can I travel with orthodontic miniscrews?	3 ± 0.82	3 ± 0.82	3 ± 0.82	20 ± 2,45	20,75 ± 3,77	21,25 ± 3,59
22	How often will I need to see my orthodontist with orthodontic miniscrews?	3.5 ± 0.58	3.25 ± 0.5	3.25 ± 0.96	22,25 ± 0,96	22,25 ± 0,96	22 ± 1,15
23	What materials are orthodontic miniscrews typically made of?	4.5 ± 0.58	4.5 ± 0.58	4.5 ± 0.58	20 ± 2,94	19 ± 2,94	20,5 ± 4,51
24	Are orthodontic miniscrews visible or do they affect the appereance of my smile?	4 ± 0	4 ± 0	4 ± 0	21,25 ± 3,59	21,25 ± 2,87	22,25 ± 4,19
25	Do orthodontic miniscrews affect the overall cost of orthodontic treatment?	3.25 ± 0.96	3.25 ± 0.96	3.25 ± 0.96	23 ± 1,41	23 ± 1,41	22,75 ± 2,22
26	Can I feel the orthodontic miniscrews in my mouth , or are they noticeable?	3.5 ± 1	3.5 ± 1	3.5 ± 1	19 ± 2,58	19 ± 2,45	17,75 ± 3,69
27	How are orthodontic miniscrews different from traditional braces and other orthodontic appliances?	4.25 ± 0.5	4 ± 0	4 ± 0	15,5 ± 4,04	16,25 ± 4,27	16 ± 4,55
28	Are there any potential long -term effects or complications associated with orthodontic miniscrews?	4 ± 0	3.75 ± 0.5	3.75 ± 0.5	20,75 ± 2,5	22 ± 3,27	21,25 ± 3,3
29	What is the success rate of orthodontic miniscrews in orthodontic cases?	4 ± 0	4.25 ± 0.5	4.25 ± 0.5	22 ± 1,41	21,75 ± 1,26	22,75 ± 0,96
30	Do orthodontic miniscrews affect speech or pronunciation?	4 ± 0	3.5 ± 1	3.5 ± 1	22 ± 1,63	22 ± 1,41	22,75 ± 1,5

The websites were evaluated simultaneously by three orthodontic residents. Three orthodontic residents, BBY, RA, and İEŞ, respectively, queried the ChatGPT, Google Bard, and Bing Chat chatbots. To prevent AI learning, a new chat was initiated for each question. These same three residents subsequently evaluated the chatbot responses using a five-point modified Likert scale: (5) the device responded with evidence-based information; (4) the device responded with adequate information; (3) the device responded but did not provide adequate information; (2) the device responded with the wrong answer; and (1) the device did not know the response to the question. The Likert scale, in which (1) represents the worst score,^15^ was employed to evaluate the adequacy and usability of the chatbot responses from a practical perspective, focusing on the completeness and accuracy of the information provided. On the other hand, the DISCERN evaluation was used to specifically assess the reliability of the information provided by the chatbots. The responses provided by LLMs were evaluated by the same three residents for reliability using the DISCERN scoring system, which is commonly employed in critical appraisal of health-related internet sources to assess their reliability.^29^ DISCERN consists of three sections and 16 questions in total. The first section assesses reliability and comprises eight questions, the second section evaluates the quality of information regarding treatment options with seven questions, and the third section determines the overall rating of the publication with one question. Scoring is conducted on a scale of 1-5 for each question, with “5” indicating meeting criteria and “1” indicating not meeting criteria (“5” indicates that it meets the criteria, and “1” indicates that no criteria are met). However, in this study, the DISCERN section 1 was modified to assess the reliability of chatbot responses, as sections 2 and 3 of DISCERN are not applicable to all 30 questions selected for the study (Table 2). The DISCERN first part tool has a maximum score of 40 and a minimum score of 8.

**Table 2:** Contents of mDISCERN Section 1.

mDISCERN criteria	Total score (8-40 points)
1. Are the aims clear?	1-5 point
2. Does it achieve its aims?	1-5 point
3. Is it relevant?	1-5 point
4. Is it clear what sources of information were used to compile the publication (other than the author or producer)?	1-5 point
5. Is it clear when the information used or reported in the publication was produced?	1-5 point
6. Is it balanced and unbiased?	1-5 point
7. Does it provide details of additional sources of support and information?	1-5 point
8. Does it refer to areas of uncertainty?	1-5 point

## STATISTICAL ANALYSIS

The data were analyzed using IBM SPSS V23. The conformity to normal distribution was examined using the Shapiro-Wilk test. Kruskal-Wallis test was used for comparing scores that did not conform to normal distribution among devices, and multiple comparisons were conducted using the Dunn test. Spearman’s Rho correlation coefficient was used to examine the relationship between continuous parameters that did not follow a normal distribution. The Interclass Correlation Coefficient (ICC) was used to examine interobserver agreement. The significance level was set at p<0.050.

## RESULTS

When interobserver reliability was analyzed for Likert, ChatGPT-3.5 (ICC= 0.700; p<0.001) and GPT-4 (ICC= 0.598; p<0.001) showed moderate agreement; Google Bard (ICC= 0.786; p<0.001) and Bing Chat (ICC= 0.794; p<0.001) showed statistically good agreement among the scores given by the three residents. When interobserver reliability was analyzed for mDISCERN ChatGPT-3.5 (ICC=0.972; p<0.001), GPT-4 (ICC=0.964; p<0.001), Google Bard (ICC=0.975; p<0.001) and Bing Chat (ICC=0.931; p<0.001) showed statistically good agreement among the scores given by the three residents. (Table 3). Additionally, intraobserver reliability scores were evaluated by three investigators for both Likert (0.897, 0.88, 0.89 respectively) and mDISCERN (0.901, 0.893, 0.872 respectively), and high levels of agreement were observed (Table 4).

[Table t3]

**Table 3: t3:** Inter-observer reliability scores.

	Likert	DISCERN	
	ICC (95% CI)	ICC (95% CI)	p
ChatGPT-3.5	0.700 (0.529 - 0.831)	0.972 (0.949 - 0.986)	<0.001
ChatGPT-4	0.598 (0.397 - 0.764)	0.964 (0.934 - 0.982)	<0.001
Bing Chat	0.794 (0.652 - 0.892)	0.931 (0.873 - 0.965)	<0.001
Google Bard	0.786 (0.65 - 0.883)	0.975 (0.953 - 0.987)	<0.001

ICC: Interclass coefficient. p<0.001.

**Table 4:** Intraobserver reliability scores.

		ICC (95% CI)	p
Likert	Investigator I	0.897 (0.791 - 0.98)	<0.001
	Investigator II	0.88 (0.799 - 0.942)	<0.001
	Investigator III	0.89 (0.812 - 0.949)	<0.001
Discern	Investigator I	0.901 (0.826 - 0.957)	<0.001
	Investigator II	0.893 (0.815 - 0951)	<0.001
	Investigator III	0.872 (0.789 - 0.936)	<0.001

ICC (95% CI): Intra-class coefficient.

Despite not providing sources in every response, the highest mean score was obtained by GPT-4 (3.84), followed by Google Bard (3.78) and GPT-3.5 (3.68). Among all bots, Bing Chat had the lowest mean score (3.37) (Table 5). It was observed that Bing Chat had significantly lower scores compared to GPT-4 and Bard for Investigators 1 and 3, and for Investigator 2 it had significantly lower scores compared to the other three bots. For all investigators, the median score values were found to be 4 for Chat GPT-3.5, 4 for GPT-4, 3 for Bing Chat, and 4 for Google Bard (Table 6).

**Table 5:** Descriptive statistics of Likert.

	Tool			
	ChatGPT-3.5	ChatGPT-4	Bing Chat	Google Bard
Mean	3.68	3.84	3.37	3.78
SD	0.58	0.47	0.78	0.68
Median	4.00	4.00	3.00	4.00
Minimum	2.00	2.00	2.00	2.00
Maximum	4.00	5.00	5.00	5.00

Statistically significant differences were found between the median score values given to the questions by all three residents, depending on the LLMs used (p = 0.016; p<0.001 and 0.017, respectively) (Table 6). Significant differences were found between the scores given by Investigator 1 and Investigator 3 to ChatGPT-4 and Google Bard and the scores given to Bing Chat. Additionally, Investigator 2 showed significant differences in the scores given to Bing Chat compared to ChatGPT-3.5 (p=0.018). No significant differences were found among the scores given to ChatGPT-3.5, GPT-4, and Bard by all investigators. (Tables 7, 8 and 9) For Investigator I, Bing Chat had a significantly lower mDISCERN score compared to ChatGPT-4 and Google Bard, while for Investigator II Bing Chat had a significantly lower mDISCERN score compared to ChatGPT-4. When comparing the four LLMs in terms of reliability, ChatGPT-4 obtained the highest mDISCERN score (23.43 ± 2.89), followed by Google Bard (23.47 ± 3.2), ChatGPT-3.5 (22.62 ± 2.97), and Bing Chat (21.63 ± 2.43), respectively (Table 10). There was a statistically significant difference in the mean mDISCERN scores among LLMs (p=0.006). There was no statistically significant relationship between evaluators’ mDISCERN and Likert scores for all LLMs (p>0.050).

**Table 6:** Comparison of numerical rating scale values according to Likert.

		Tool				Test Statistic	p*
		ChatGPT-3.5	ChatGPT-4	Bing Chat	Google Bard		
Investigator I	Mean ± SD	3.7 ± 0.6	3.83 ± 0.53	3.44 ± 0.7	3.8 ± 0.71	10.367	0.016
	Median (min-max)	4 (2 - 4)^ab^	4 (2 - 5)^a^	3 (2 - 5)^b^	4 (2 - 5)^a^		
Investigator II	Mean ± SD	3.7 ± 0.53	3.87 ± 0.35	3.23 ± 0.77	3.73 ± 0.74	18.904	<0.001
	Median (min-max)	4 (2 - 4)^a^	4 (3 - 4)^a^	3 (2 - 5)^b^	4 (2 - 5)^a^		
Investigator III	Mean ± SD	3.63 ± 0.61	3.83 ± 0.53	3.43 ± 0.86	3.8 ± 0.61	10.239	0.017
	Median (min-max)	4 (2 - 4)^ab^	4 (2 - 5)^a^	3 (2 - 5)^b^	4 (2 - 5)^a^		

*Kruskal- Wallis test; a-b: There is no difference between tools with the same letter.

SD = Standard Deviation. *p<0.05

**Table 7:** Comparison results between tools for Investigator I.

	Likert		DISCERN	
Tools	Test Statistic	p	Test Statistic	p
Bing Chat-ChatGPT-3.5	15.261	0.239	14.95	0.556
Bing Chat-ChatGPT-4	20.711	0.032	30.583	0.003
Bing Chat -Google Bard	-21.011	0.028	-23.867	0.044
ChatGPT-3.5-ChatGPT-4	-5.45	1.000	-15.633	0.472
ChatGPT-3.5- Google Bard	-5.75	1.000	-8.917	1.000
ChatGPT 4- Google Bard	-0.3	1.000	6.717	1.000

*Kruskal-Wallis test. *p<0.05.

**Table 8:** Comparison results between tools for Investigator II.

	Likert		DISCERN	
Tools	Test Statistic	p	Test Statistic	p
Bing Chat-ChatGPT-3.5	22.733	0.018	11.833	1.000
Bing Chat-Google Bard	-25.867	0.004	-21.417	0.098
Bing Chat-ChatGPT-4	30.6	<0.001	27.35	0.013
ChatGPT-3.5- Google Bard	-3.133	1.000	-9.583	1.000
ChatGPT-3.5-ChatGPT-4	-7.867	1.000	-15.517	0.490
Google Bard -ChatGPT-4	4.733	1.000	5.933	1.000

*Kruskal-Wallis test. *p<0.05.

**Table 9:** Comparison results between tools for Investigator III.

	Likert		DISCERN	
Tools	Test Statistic	p	Test Statistic	p
Bing Chat-ChatGPT-3.5	12.683	0.613	11.933	1.000
Bing Chat-Google Bard	-21.1	0.039	-22.317	0.074
Bing Chat-ChatGPT-4	21.75	0.030	22.217	0.076
ChatGPT-3.5- Google Bard	-8.417	1.000	-10.383	1.000
ChatGPT-3.5-ChatGPT-4	-9.067	1.000	-10.283	1.000
Google Bard -ChatGPT-4	0.65	1.000	-0.1	1.000

*Kruskal-Wallis test. *p<0.05.

**Table 10:** Comparison of numerical rating scale values according to mDISCERN.

	Tool				Test Statistic	p*
	ChatGPT-3.5	ChatGPT-4	Bing Chat	Google Bard		
Investigator I	22.43 ± 2.87	23.27 ± 2.89	21.4 ± 2.24	23.33 ± 3.37	13.266	0.004
	22.5 (13 - 28)ab	24 (12 - 29)^a^	22 (16 - 25)^b^	23 (16 - 30)^a^		
Investigator II	22.6 ± 3.04	23.57 ± 2.99	21.63 ± 2.59	23.33 ± 3.12	10.79	0.013
	23 (13 - 29)ab	24 (13 - 28)^b^	22 (16 - 26)^a^	24 (16 - 29)^ab^		
Investigator III	22.83 ± 3.23	23.47 ± 3.09	21.87 ± 2.91	23.73 ± 3.35	8.478	0.037
	23 (13 - 29)	24 (12 - 28)	22 (13 - 26)	24 (15 - 29)		
	22.62 ± 2.97	23.43 ± 2.89	21.63 ± 2.43	23.47 ± 3.2	12.394	0.006
	22.5 (13 - 28.33)^ab^	23.83 (12.33 - 28.33)^a^	21.67 (15 - 25.33)^b^	23.33 (16 - 28.33)^a^		

* Kruskal-Wallis Test a-b: There is no difference between tools with the same letter.

## DISCUSSION

Artificial Intelligence (AI) has influenced our daily lives by designing and evaluating advanced applications and devices capable of performing various functions. A chatbot is an AI program and a Human-Computer Interaction (HCI) model.^30^ The term ‘chatbot’ is defined in the dictionary as, “A computer program designed to respond with conversational or informational replies to verbal or written messages from users.” (Chatbot |Definition of chatbot in English by Oxford Dictionary (Lexico), 2023).

ChatGPT is an advanced artificial intelligence language model that was first launched by OpenAI in November 2022. It is claimed to be better than other LLMs in generating human-like text and maintaining consistency in a conversation.^5^ ^,^ ^31^ With the increasing popularity of chatbots today, it’s observed that Google Bard and Bing Chat, like ChatGPT, are featured in academic studies in the health field.^11^ The easy accessibility of bots makes them the best option for many users.^6^

TADs have become devices used by contemporary orthodontists during routine clinical practices as a direct anchorage unit or connected to a dental anchorage unit indirectly to prevent unwanted tooth movement.^32^ TADs are also frequently used in clear aligner cases, which brought a different dimension to the aesthetic perception of orthodontists and patients with their introduction to the market.^33^ In cases involving movements that are difficult to achieve with clear aligners, TAD indications exist. For example, cases planned for the retraction of both maxillary and mandibular anterior teeth with maximum anchorage, distalization of mandibular molars, and correction of severe deepbite can be treated as required owing to TADs.^34^ Miniscrews are also used in the miniscrew-assisted rapid palatal expansion (MARPE) treatment method, which maximizes skeletal effects and minimizes dentoalveolar effects, in patients with maxillary transverse deficiency.^35^

Brandão et al.^36^ assessed the acceptance and compliance of TADs by patients and found that about one-fourth of the patients were anxious before the procedure. Despite most patients trusting their orthodontists and accepting treatment with miniscrews, their need for more information was noteworthy. The main concerns of patients were the duration of the surgery and the placement method, followed by the advantages of using miniscrews and their sizes. As seen in this study, TADs, which have undeniable clinical importance, are a subject of curiosity for patients.

The ability for patients to access enlightening and accurate information online about topics they are curious and concerned about could facilitate the acceptance of treatment and prevent time loss during appointments. Abu Arqub et al.^27^ recently evaluated the content of answers given by artificial intelligence (ChatGPT) about orthodontic clear aligners and found that only 58% of the responses were objectively true. The reliability and evidence-based response capacity of the answers regarding clear aligners were found to be low. Additionally, it was noted that the answers were not at an expert level. Although there has been debate about AI chatbots’ tendency to make assumptions when insufficient information is provided, which can lead to unwanted consequences in medical topics,^5^ there has been no study to date evaluating the reliability of these four LLMs in the field of orthodontic miniscrews. This study aimed to evaluate the performance of the mentioned applications in educating patients, considering they could provide significant help.

In this study, the orthodontic residents who queried the chatbots also evaluated the responses. This approach was chosen to leverage their knowledge and ensure a consistent understanding of the questions and responses. While this dual role might introduce potential evaluator bias, we mitigated this risk by using multiple evaluators and assessing interobserver and intraobserver reliability statistically (e.g., ICC). The strong agreement among evaluators supports the validity of these findings.

Another important point in this study is that the four LLMs were examined by three residents to assess more accurately whether there were differences in the responses given by the LLMs. The Likert scale, with a score of (1) representing the lowest rating, was employed to evaluate the adequacy and usability of the chatbot responses, with particular emphasis on the completeness and accuracy of the information provided. In contrast, the DISCERN evaluation was utilized to specifically assess the reliability of the information delivered by the chatbots. Looking at the findings, it is determined that Bing Chat’s responses to Investigator 2 were more inconsistent and incomplete. Consistent with this study, Perez-Pino et al.^15^ also drew attention to significant differences in the responses given to researchers by AI-based virtual assistants. When evaluating the results of this study, it appears that GPT-4 provided more accurate answers to popular orthodontic miniscrew questions asked by patients compared to other LLMs. In a study by Massey et al.^7^ on orthopedic assessment examinations, GPT-4 was found to be better than ChatGPT-3.5, similar to this research. In addition, a study evaluating ChatGPT-3.5, GPT-4, and Google Bard in generating differential diagnoses in clinicopathological conferences of neurodegenerative disorders found that the percentages of correct diagnoses made by the bots were 32%, 52%, and 40%, respectively, supporting this study.^8^ Another study with results consistent with this one was conducted by Gan et al.^31^, evaluating the performance of Google Bard and ChatGPT in the triage of mass casualty incidents. Google Bard was found to be superior to ChatGPT in correctly performing mass casualty triage, with significant differences in accuracy between Google Bard (60%) and ChatGPT (26.67%) (p = 0.002). The results obtained should be noted as reflecting the different strengths of LLMs in various fields. Consistent with the findings of this study, Patil et al.^4^ also demonstrated that GPT-4 performed better than Bard. In their study evaluating responses to radiology board exam practice questions, the accuracy percentages were 87.11% vs 70.44%, respectively. As in this study, it was found that both chatbots consistently provided explanations for their answers, regardless of whether the answer was correct or incorrect. The superior performance of GPT-4 over Bard is likely due to GPT-4 having a wider range of learning data sets and a greater ability to answer medical questions.^4^ In the study by Rahsepar et al.^11^, which evaluated the accuracy of answers provided to frequently asked lung cancer questions by ChatGPT-3.5, Google Bard, Bing Chat, and Google search, and in the recent study by Ali et al.^10^, which assessed the performance of ChatGPT-3.5, GPT-4, and Google Bard on a question bank designed specifically for neurosurgery oral boards examination preparation, ChatGPT-3.5 showed higher performance than Google Bard, contrary to our study (p= 0.004 and p<0.01, respectively). In our study, the reason for Google Bard’s higher performance over ChatGPT-3.5 is likely due to its ability to provide detailed and accurate answers, along with having live internet access (open-source resources) to include references in its responses, and ChatGPT’s inability to access current information after January 2022. Considering that the DISCERN first section tool has a maximum score of 40 and a minimum score of 8, if a mean score of 24 is accepted, it can be said that the reliability of the responses provided by chatbots are below the mean value. Similar to this study, Seth et al.^37^ compared the effectiveness of LLMs in providing information about rhinoplasty in their study. They found that GPT-4 and Google Bard had higher mDISCERN scores compared to ChatGPT-3.5 and especially Bing Chat.

The reason for Bing Chat, supported by GPT-4 technology and also providing open-source resources, receiving the lowest score among LLMs is its tendency to give very short, insufficient, and sometimes incomplete responses. Giannakopoulos et al.^6^ also found Bing Chat’s performance to be lower than others, consistent with this study. Despite the advantages of Bing Chat having live internet access and access to GPT-4, its limited accessibility is a disadvantage. Bing Chat’s daily conversation limit is 100 requests, which is quite restrictive compared to ChatGPT’s 70 requests per hour.^6^ However, this limitation did not impact its performance in this study.

One potential limitation of this study is the lack of blinding of the chatbots prior to evaluation. The literature presents two approaches: some studies employ blinding^38^, while others do not^15^. Although we did not employ blinding in our study, we took several measures to mitigate potential bias. The evaluators were not informed of which large language model (LLM) might perform better and held no prior expectations or knowledge. Their assessments were based solely on the quality and content of the chatbot responses, evaluated using the predefined criteria of the Likert scale and the mDISCERN tool. Additionally, the strong intraobserver agreement demonstrated by the Intraclass Correlation Coefficient (ICC) further supports the consistency and reliability of the evaluations, minimizing the impact of any potential bias in the assessment process.

A second limitation of this study is the lack of evaluation of changes in responses over time. Previous studies, such as the one by Goodman et al.^39^, have shown that incorrect answers were reintroduced and reevaluated, resulting in improved outcomes. However, we did not perform this step in our study because we had relatively few incomplete responses, and re-evaluating them was not deemed necessary. It is important to note that time-related changes in chatbot responses could reveal variations in accuracy and reliability, as highlighted in studies on ChatGPT. Therefore, the reliability of chatbot responses should be supported by professional oversight to ensure consistency and accuracy over time. 

While this study did not directly evaluate the clinical consequences of low-quality responses provided by ChatGPT-3.5 and Bing Chat, it is important to consider their potential impact. Inaccurate or incomplete information might mislead patients, potentially increasing anxiety, delaying appropriate treatment, or fostering unrealistic expectations. This highlights the importance of complementing chatbot-generated information with guidance from qualified healthcare professionals. Future studies could focus on exploring the real-world implications of chatbot use in patient decision-making to better understand these risks.

## CONCLUSION

The Likert and mDISCERN scores of ChatGPT-4 and Google Bard were found to be higher compared to ChatGPT-3.5 and Bing Chat. Although the responses provided by these applications regarding orthodontic miniscrews are sufficient for patients to gain general knowledge, they should always be supplemented with information provided by professionals.
